# Binding of TGF-β1 latency-associated peptide (LAP) to αvβ6 integrin modulates behaviour of squamous carcinoma cells

**DOI:** 10.1038/sj.bjc.6600545

**Published:** 2002-10-07

**Authors:** G J Thomas, I R Hart, P M Speight, J F Marshall

**Affiliations:** Department of Oral Pathology, Eastman Dental Institute, University College London, UK; Richard Dimbleby/Cancer Research UK Laboratories, GKT School of Medicine, St Thomas' Hospital, London SE1 7EH, UK

**Keywords:** integrins, squamous carcinoma, MMP-9, TGF-β1, LAP, adhesion, migration, invasion

## Abstract

The integrin αvβ6 is not detectable on normal keratinocytes *in vivo* but expression is increased significantly in oral squamous cell carcinoma where this heterodimer has been shown to play a role in cell migration, invasion and protease expression. Although regarded initially as a fibronectin receptor, αvβ6 may bind to arginine-glycine-aspartic acid sequences in other matrix molecules including tenascin and vitronectin. Interestingly, αvβ6 has also been shown to have high affinity for the TGF-β1 latency associated peptide and to participate in the activation of the TGF-β1 latent complex. Since TGF-β1 is present in squamous carcinomas, it is possible that latency associated peptide may modulate malignant keratinocyte behaviour independently from the classical TGF-β signalling pathways through its interaction with integrins. We show here that when latency associated peptide is immobilised onto a surface, it acts as an αvβ6-specific ligand for oral squamous carcinoma cells promoting adhesion and haptotactic migration in addition to αvβ6-dependent increase in pro-MMP-9 expression. In contrast, even very low concentrations of soluble latency associated peptide (0.1 μg ml^−1^) inhibited αvβ6-dependent adhesion, migration and invasion. Thus αvβ6-dependent processes of oral squamous cell carcinoma, is likely to be modulated, not only by the local concentration of latency associated peptide in the stroma, but also whether it is immobilised in the matrix or released as a soluble protein.

*British Journal of Cancer* (2002) **87**, 859–867. doi:10.1038/sj.bjc.6600545 www.bjcancer.com

© 2002 Cancer Research UK

## 

TGF-β belongs to a multifunctional cytokine family composed of three highly homologous genes, *TGFB1*, *TGFB2* and *TGFB3* that encode polypeptides with similar biological functions (Massague *et al*, 2000). TGF-β is unusual among the known cytokines as it is secreted as a latent complex and is found *in vivo* primarily in the latent form (Gleizes *et al*, 1997). The TGF-β isoforms are synthesised as dimeric precursor proteins, which are cleaved during secretion to yield mature cytokines. The mature TGF-β remains associated with its propeptide (the latency-associated peptide-LAP) as the so-called small latent complex (SLC) from which TGF-β must be released to elicit its biological activity (Gleizes *et al*, 1997). More commonly, the SLC may further associate with members of another protein family, the latent TGF-β-binding proteins (LTBPs) forming the large latent complex (LLC) (Gleizes *et al*, 1997). The LLC associates with matrix fibrils and may be incorporated covalently into the extracellular matrix by cross-linking of LTBP-1 to matrix proteins (Taipale *et al*, 1996).

LAP-β1 and LAP-β3 isoforms contain an arginine-glycine-aspartic acid (RGD) sequence, which is also present in a number of extracellular matrix molecules where it acts as a binding sites for integrins (Munger *et al*, 1998). Investigation of latent TGF-β1 as a possible integrin ligand led to the discovery that A549 lung adenocarcinoma cells adhere to LAP-β1 using αvβ1 and αvβ5 integrins (but migrate on LAP using αvβ1) (Munger *et al*, 1998). This study demonstrated that the avidity of αvβ1 binding was greater for unbound LAP than when it was complexed as the SLC or LLC, raising the possibility that integrin binding to LAP may generate signals independent of classical TGF-β signalling. More recent observations using β6 transfected colon adenocarcinoma cells revealed that LAP is a high affinity ligand for αvβ6 and that the interaction of LAP with αvβ6 induces phosphorylation of downstream components of integrin-signalling complexes. Furthermore αvβ6 may provide a mechanism for the activation of latent TGF-β1 (Munger *et al*, 1999). Similar to adenocarcinoma cells, keratinocytes also activate TGF-β1 through the interaction of αvβ6 with LAP (Munger *et al*, 1999). However, the effect of this protein in modulating keratinocyte behaviour, particularly cell movement, through integrin interactions has not been investigated.

The integrin αvβ6 is expressed primarily on epithelial cells and binds to RGD sites in fibronectin, vitronectin and tenascin (as well as LAP) (Busk *et al*, 1992; Prieto *et al*, 1993; Huang *et al*, 1998). Expression of αvβ6 is not detectable on keratinocytes in adult oral epithelium or epidermis. However, several studies have found high expression of αvβ6 in oral squamous cell (and other carcinomas), suggesting that this integrin may play a role in tumour progression (Breuss *et al*, 1995; Jones *et al*, 1997; Arihiro *et al*, 2000; Hamidi *et al*, 2000; Ahmed *et al*, 2002).

The αvβ6 heterodimer has been shown to modulate several processes in keratinocytes, including migration on fibronectin and vitronectin, and also fibronectin-dependent upregulation of matrix metalloproteinase-9 (MMP-9) (Huang *et al*, 1998; Thomas *et al*, 2001a). In addition to promoting cell migration and MMP-9 upregulation, we have demonstrated that increased expression of αvβ6 in squamous cell carcinoma cell lines produces a more invasive phenotype; such increased aggressive behaviour is αvβ6-dependent and is modulated through altered protease expression (Thomas *et al*, 2001b,c). A recent study has also shown that the progression of oral squamous carcinoma *in vivo* can be significantly retarded using inhibitory anti-αvβ6 antibodies (Xue *et al*, 2001).

TGF-β is a potent growth inhibitor in normal epithelial tissues and for this reason considerable emphasis has been given to the concept that TGF-β is highly protective against cancer, and that genetic or epigenetic loss of TGF-β signalling leads to tumour outgrowth and progression (Derynck *et al*, 2001; Wakefield and Roberts, 2002). In addition to growth inhibition, recent work has implicated TGF-β in other processes involved in tumour inhibition including maintenance of genomic stability, induction of senescence, suppression of telomerase activity and prevention of inappropriate angiogenesis (Wakefield and Roberts, 2002). However, the role of TGF-β in tumour biology is complex involving several signalling pathways, and a number of studies have demonstrated that TGF-β1 may be pro-oncogenic, driving malignant progression, invasion and metastasis (Akhurst and Derynck, 2001; Wakefield and Roberts, 2002). For example, recent data suggests that aberrant activation of MAPK pathways may play an important role in diverting the TGF-β response towards a pro-oncogenic outcome, and that TGF-β and activated Ras may cooperate to promote invasive, metastatic disease (Park *et al*, 2000). It is now suggested that TGF-β has biphasic effects during tumorigenesis, initially acting as a tumour suppressor, but later stimulating cancer progression (Akhurst and Balmain, 1999; Akhurst and Derynck, 2001).

With regard to cancer development in keratinocytes the majority of studies have focused on the role of TGF-β1 in animal models of skin carcinogenesis (Akhurst and Balmain, 1999). These have also demonstrated that TGF-β appears to have biphasic actions on tumour cells, having an important dominant negative growth effect at early stages, but at later stages enhancing the malignant conversion rate and invasion through epigenetic mechanisms. A number of studies have shown that the majority of oral squamous carcinoma cell lines remain sensitive to the growth inhibitory effect of TGF-β even in the absence of Smad-4 (suggesting that Smad-4 independent pathways may also mediate growth inhibitory responses) (Paterson *et al*, 1995, 2002; Malliri *et al*, 1996). However, tumour cells may escape negative growth regulation by producing less autocrine TGF-β1, expressing low levels of the receptor TβR-II, or losing expression of Smad-2 (Fahey *et al*, 1996; Muro-Cacho *et al*, 2001; Paterson *et al*, 2001). Structural defects in the TβR–I or TβR-II receptor have also been reported in oral cancers although these are not common, suggesting that alterations of gene expression rather than gene mutation are likely to be more important in the pathogenesis of oral cancer (Garrigue-Antar *et al*, 1995; Wang *et al*, 1997; Paterson *et al*, 2001). TGF-β1 is frequently detected in squamous cell carcinomas (Eisma *et al*, 1996; Akhurst and Balmain, 1999) and it is likely that, independently of tumour cell responses to classical TGF-β1 signalling, the presence of LAP in the tumour stroma may modulate malignant cell behaviour through its interaction with integrins.

We have investigated the potential of LAP to modulate oral SCC behaviour. We report that surface-immobilised LAP, which functions exclusively as an αvβ6-dependent ligand in keratinocyte-derived cells, promotes adhesion, migration and increased pro-MMP-9 secretion. In contrast, soluble LAP mediates αvβ6-specific inhibition of adhesion, migration and invasion. Thus, independently of classical TGF-β signalling, the response of oral SCC cells to TGF-β *in vivo* is likely to be determined, not only by the local concentration of LAP but also by the ratio of immobilised to soluble LAP.

## MATERIALS AND METHODS

### Antibodies and reagents

A series of monoclonal antibodies (mAbs) (all of murine origin) was used in this study. Anti-human αv (L230; Weinacker *et al*, 1994) was prepared in our laboratory from hybridoma cells obtained from the American Type Culture Collection (Rockville, MD, USA). The anti-αvβ6 (E7P6 and R6G9; Weinacker *et al*, 1994), anti-αvβ6 (10D5; Huang *et al*, 1998), anti-α5β1 (P1D6) and anti-β1 (P4C10) antibodies were purchased from Chemicon International (Harrow, UK). Anti-αvβ5 (P1F6) was obtained from Life Technologies, Paisley, UK. W632 (anti-MHC class I) was a kind gift from W Bodmer (IMM, Oxford). FITC- and horseradish peroxidase-conjugated rabbit anti-mouse antibodies were purchased from Dako (High Wycombe, UK). Plasma fibronectin, TGF-β1 latency associated peptide (LAP) and BSA were purchased from Sigma Chemical Co (Poole, Dorset, UK).

### Cell cultures

Using cDNA transfection techniques we previously created a panel of cell lines expressing various levels of αvβ6 (Thomas *et al*, 2001b). H357 is an αv-negative oral squamous carcinoma cell line (Prime *et al*, 1990; Sugiyama *et al*, 1993) from which the V3 cell line was generated by transfection of αv cDNA (Jones *et al*, 1996); V3 cells predominantly express the αvβ5 heterodimer. This cell line was retrovirally infected with β6 cDNA creating the VB6 cell line, which has high αvβ6 expression. A null transfectant control cell line for the VB6 cells (C1) was also generated at this time (Thomas *et al*, 2001b). In light of the report of Park *et al* (2000) showing the co-activity of activated Ras and TGF-β, it is perhaps worth noting that H357 cells, and the derivative cell lines described above, all retain sensitivity to the growth inhibitory effects of TGF-β (Paterson *et al*, 1995; data not shown) despite the fact that H357 cells contain c-Ha-ras gene point mutations at the activating codons 13 and 61 (Yeudall *et al*, 1993).

Cells were grown in standard keratinocyte growth medium (KGM) as described (Sugiyama *et al*, 1993; Jones *et al*, 1996). KGM comprised α-MEM containing 10% foetal calf serum (Globepharm, Surrey) supplemented with 100 IU l^−1^ penicillin, 100 μg l^−1^ streptomycin and 2.5 μg l^−1^ amphotericin B (Gibco BRL), 1.8×10^−4^ M adenine, 5 μg ml^−1^ insulin, 1×10^−10^ M cholera toxin, 0.5 μg ml^−1^ hydrocortisone and 10 ng ml^−1^ epidermal growth factor (Sigma). All cells were tested routinely for mycoplasma.

### Flow cytometry

Subconfluent cells were washed twice with PBS and harvested by trypsin/EDTA (0.25% w v^−1^, 5 mM). Cells were washed once in PBS containing 10% FCS. Cells were incubated with primary antibody for 40 min at 4°C and washed twice with PBS. FITC-conjugated secondary antibody was applied to the cells for 30 min at 4°C. Briefly, cells were washed twice with PBS and resuspended in 0.5 ml PBS with 10% FCS. Labelled cells were scanned on a FACSCalibur cytometer (Becton-Dickinson) and analysed using Cellquest software, acquiring 1×10^4^ events.

### Adhesion assays

A 50 μl solution of LAP at a concentration of 0.25 μg ml^−1^ was added to 96-well plates (Falcon 3912; Becton Dickinson) and incubated at 37°C for 1 h. After incubation wells were washed with PBS then blocked with 0.1% BSA at 37°C for 30 min. Control wells were incubated with 0.1% BSA. Cells were chromium [^51^Cr] labelled (Brunner *et al*, 1976) and resuspended in PBS containing 2.5 mM EDTA for 10 min. Cells were washed and resuspended in cold α-MEM (1.5×10^4^ cells per well). For blocking experiments, cells were incubated with specific antibodies (as described in Results) for 10 min on ice in each well. Plates were incubated at 37°C for 30 min. Non-adherent cells were removed by flooding plates with PBS supplemented with 1 mM CaCl_2_ and 0.5 mM MgCl_2_. After two washes, the plates were cut into individual wells and the radioactivity associated with each well was determined in a gamma counter (1261 Multigamma; LKB Wallac, Bromma, Sweden). The per cent adhesion was expressed as the adherent cell radioactivity as a proportion of the total cell input. The non-specific adhesion (attachment to wells coated with BSA) was subtracted. Experiments were repeated on six occasions in quadruplicate, with similar results.

### Preparation of cell supernatants for MMP determination

Twenty-four-well plates were coated with fibronectin (10 μg ml^−1^) or LAP(0.25 μg ml^−1^). A 200 μl solution was added to the wells and incubated at 37°C for 1 h. After incubation wells were washed with PBS and blocked with 0.1% BSA for 30 min. 10^5^ cells in α-MEM were seeded into each well. For blocking experiments, cells were incubated with anti-αvβ6 antibody (10D5) or an irrelevant control antibody (W632; anti-MHC CLASS 1) for 30 min at 4°C and plated in medium containing an excess of antibody. Supernatant was sampled after 24 h at which time a cell count was carried out. Conditioned medium was cleared of cells and debris by centrifugation at 4000 r.p.m. for 10 min.

### Zymography

MMP-9 activity was analysed using SDS–PAGE-substrate gels. Gelatin (bloom 300, Sigma) was added to a 12% acrylamide separating gel at a final concentration of 1 mg ml^−1^. To each gel, supernatant samples containing equal volume/cell number were mixed with non-reducing sample buffer (62.5 mM Tris-HCl, pH 6.8, 10% glycerol, 2% SDS, 0.1% bromophenol blue) and added to the gel without boiling. MMP-9 standard was run on each gel. Following electrophoresis, gels were washed twice in 2.5% Triton X-100 for 30 min at 37°C to remove the SDS. Gels were incubated at 37°C overnight in developing buffer containing 50 mM Tris-HCl, 0.2 M NaCl, 5 mM CaCl_2_ and 0.02% Triton X-100. Gels were stained with 0.5% Coomassie blue G250 in 30% methanol, 10% glacial acetic acid for 30 min and destained in the same solution without Coomassie blue. Gelatin-degrading enzymes were identified as clear bands against the blue background of the stained gel. Images of stained gels were captured under illumination using the UVP Imagestore 5000 (Ultra-Violet Products UK) and exported for use on a PC using the Scion image program (Scion Corp; based on the Macintosh NIH Image program developed at the National Institutes of Health, USA). The intensity of the bands was measured by densitometric analysis and comparisons made within each gel to determine relative changes in MMP activity. Data for each zymogram were expressed as relative changes in MMP activity and these relative changes compared with repeat experiments. Experiments were repeated a minimum of three times in duplicate or triplicate.

### Migration assays

Haptotactic cell migration assays were performed using matrix coated polycarbonate filters (8 μm pore size, Transwell®, Beckton Dickinson). The membrane undersurface was coated with fibronectin (10 μg ml^−1^) or LAP (0.25 μg ml^−1^) in PBS for 1 h at 37°C and blocked with migration buffer (0.1% BSA in α-MEM) for 30 min at 37°C. For blocking experiments, cells were incubated with antibody for 30 min at 4°C prior to seeding. The lower chamber was filled with 500 μl of migration buffer, following which cells were plated in the upper chamber of quadruplicate wells, at a density of 5×10^4^ in 100 μl of migration buffer and incubated at 37°C for 12 h. Following incubation, the cells in the lower chamber (including those attached to the undersurface of the membrane) were trypsinised and counted on a Casy 1 counter (Sharfe System GmbH, Germany).

### Invasion assays

Cell invasion assays were performed using Matrigel coated polycarbonate filters (8 μm pore size, Transwell®, Beckton Dickinson). Matrigel (70 μl; 1 : 2 dilution in α-MEM) was added to the upper membrane and allowed to gel for 1 h at 37°C. For blocking experiments, cells were incubated with anti-integrin antibody for 30 min at 4°C prior to seeding. To act as a chemoattractant, 500 μl of KGM was placed in the lower chamber. Cells were plated in the upper chamber of quadruplicate wells at a density of 5×10^4^ in 200 μl of α-MEM and incubated at 37°C for 72 h. The cells in the lower chamber (including those attached to the undersurface of the membrane) were then trypsinised and counted on a Casy 1 counter (Sharfe System GmbH, Germany).

### Statistical analysis

Data are expressed as the mean±s.d. of a given number of observations. Where appropriate, one way analysis of variance (ANOVA) was used to compare multiple groups. Comparisons between groups were by Fishers PLSD (set at 5% significance). A *P* value of <0.05 was considered to be significant.

## RESULTS

### The VB6 cell line expresses high levels of αvβ6

Figure 1

**Figure 1:**
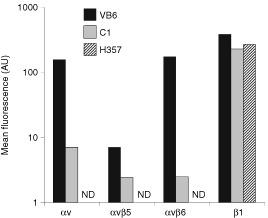
Flow cytometric analysis of integrin expression by cell lines. The geometric mean fluorescence (arbitrary units, log scale) as measured by flow cytometry of cells labelled with anti-integrin antibodies is shown. Negative control had secondary antibody only and has been subtracted from the results. Figure shows a representative experiment (ND=not detected). Flow cytometry confirmed high αvβ6 expression by VB6 cells and that C1 null transfectant cells express low levels of endogenous αvβ6. H357 cells are αv- negative. The cell lines express similar levels of β1 integrins.

confirms, as we have shown previously, that VB6 cells express approximately 50 times more αvβ6 than C1 cell control cells. These cell lines do not express αvβ1 or αvβ3 (Thomas *et al*, 2001b,c). We also confirmed that the gene transfer procedures had not altered levels of other integrins expressed at the cell surface by the cells (Thomas *et al*, 2001b). The cell lines were shown to express similar levels of β1 integrins (Figure 1). H357 is αv-negative (Figure 1).

### Adhesion to LAP is mediated through αvβ6

**Figure 2:**
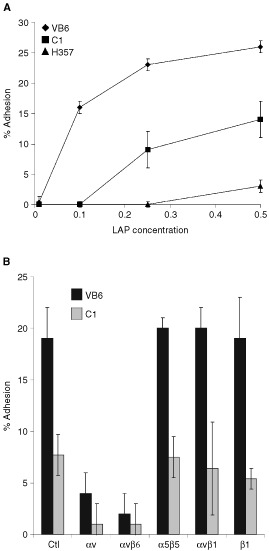
Cell adhesion to LAP is αvβ6-dependent. Chromium [^51^Cr]- labelled cells (1.5×10^4^) were added to LAP-coated 96-well plates containing an irrelevant control antibody (W632 anti-MHC class 1) or test antibodies against αv (L230), αvβ5 (P1F6), αvβ6 (10D5), β1 (P4C10) and α5β1 (P1D6). Background binding to BSA has been subtracted from the results. Figures show representative experiments performed in quadruplicate. Error bars represent standard deviation. (**A**) VB6 cells show increased adherence LAP. β6 transfected cells (VB6), control cells (C1) and αv-negative cells (H357) were plated onto varying concentrations of LAP. VB6 cells showed significantly increased adhesion compared to C1 cells (which express low levels of endogenous αvβ6). H357 cells did not adhere. (**B**) Adhesion to LAP is αvβ6-dependent. VB6 and C1 adhesion to LAP was inhibited by anti-αvβ6 antibody or anti-αv antibody. Antibodies against αvβ5, α5β1 or β1 produced no effect. These data suggest that adhesion of VB6 and C1 cells is modulated solely through αvβ6.

Preliminary studies indicated that optimal adhesion to LAP was achieved at a concentration of 0.25 μg ml^−1^ (Figure 2A

) and that the level of adhesion at this concentration was similar to that produced on a fibronectin-coating of 10 μg ml^−1^ (data not shown). The cell lines were plated onto LAP in the presence or absence of blocking antibodies against the αv subunit (L230), β1 subunit (P4C10), αvβ5 (P1F6), αvβ6 (10D5), α5β1 (P1D6) or an irrelevant antibody against MHC class 1 (W632). Over six separate experiments adhesion of VB6 cells was significantly higher (20% total adhesion) than C1 cells (8.5% total adhesion) (*P*=<0.001). Adhesion of VB6 cells was inhibited completely by the anti-αvβ6 antibody, 10D5 (Figure 2B). Lung adenocarcinoma cells have been shown previously to bind LAP using αvβ5 (Munger *et al*, 1998), however, antibodies against this integrin had no effect on VB6 adhesion (Figure 2B). Antibodies against the β1 subunit (P4C10) or α5β1 (P1D6) (Figure 2B) or other α subunits (including α2, α5, α6 and α9) (data not shown) had no effect on the level of adhesion. Although C1 cells were significantly less adhesive under these conditions, inhibition of their adherence followed a similar pattern and this activity was also inhibited completely by antibodies against αvβ6 (Figure 2B). In order to determine whether other, non-αv integrins could bind to LAP, the adhesion assay was repeated using αv negative H357 cells (Figure 2A). These cells did not adhere to LAP confirming that adhesion to LAP in the cell lines was modulated solely through αvβ6.

### Migration towards LAP is αvβ6-dependent

**Figure 3:**
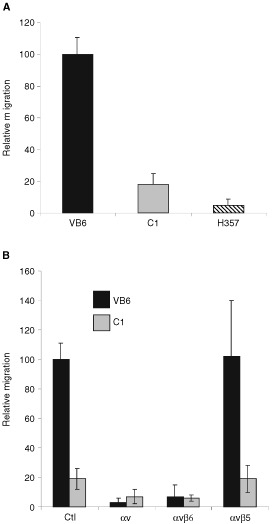
Cell migration towards LAP is αvβ6-dependent. Cells were allowed to migrate towards LAP in haptotactic migration assays. To assess integrin specificity of migration, integrin-blocking antibodies against αv (L230), αvβ5 (P1F6), αvβ6 (10D5), or a control antibody (W632) were added to VB6, C1 and H357 cells prior to plating into wells. Following 8 h incubation, the cells in the lower chamber (including those attached to the undersurface of the membrane) were trypsinised and counted on a Casy 1 counter (Sharfe System GmbH, Germany). Results for the cell lines are expressed relative to VB6 migration following incubation with an irrelevant control antibody (=100). Figures show representative experiments performed in quadruplicate. Error bars represent standard deviation. (**A**) VB6 cells show increased migration towards LAP. Comparison of migration of VB6, C1 and H357 cells towards LAP. Migration is higher in the αvβ6 expressing VB6 cells. αv-negative H357 cells did not migrate significantly. (**B**) Migration towards LAP is αvβ6-dependent. Migration of VB6 and C1 cells was inhibited completely by antibody inhibition of αvβ6 or αv. Antibodies inhibiting αvβ5 produced no effect. These data suggest that migration of VB6 and C1 cells is modulated solely through αvβ6.

VB6 cells show increased αvβ6-dependent migration towards fibronectin relative to the other cell lines (Thomas *et al*, 2001b). To determine whether VB6 cells migrated towards LAP, haptotactic migration assays were performed using LAP-coated Transwell filters. VB6 cells migrated less well towards LAP than fibronectin (a relative reduction of 53% over four experiments; *P*=0.039; data not shown). However, migration towards LAP was increased significantly in VB6 cells compared with C1 cells (*P*=<0.001; (Figure 3A

). H357 cells, which are αvβ6 negative did not migrate towards LAP and did not differ significantly from random migration towards BSA controls (Figure 3A).

The migration of VB6 cells towards LAP was abolished by antibodies against the αv subunit or αvβ6 but not by antibodies against αvβ5 (Figure 3B). These data combined with the evidence that H357 cells do not migrate towards LAP, suggest that haptotactic migration towards LAP by the various cell lines is also modulated solely through αvβ6.

### Binding of αvβ6 to LAP induces upregulation of MMP-9

**Figure 4:**
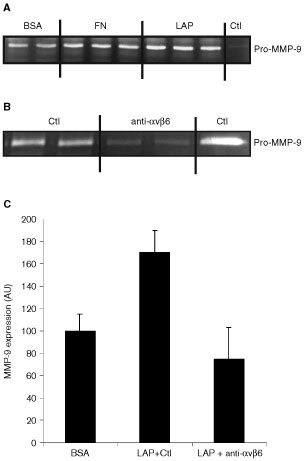
Binding of αvβ6 to LAP induces upregulation of MMP-9. Zymography for MMP-9. Cells were grown for 24 h in additive-free medium before supernatant sampling and cell counting. Samples containing equal volume per cell number were run on each gel with MMP-9 control. The intensity of the bands was measured by densitometric analysis and comparisons made within each gel to determine relative changes in MMP activity. (**A**) Zymogram showing upregulation of MMP-9 expression by VB6 cells when plated on fibronectin (FN) and LAP relative to BSA-coated plastic. Control is pro-MMP-9. (**B**) Zymogram showing the inhibition of MMP-9 expression by VB6 cells plated onto LAP following blockade with anti-αvβ6 antibody 10D5 relative to an irrelevant control antibody (Ctl; W632 (anti-MHC class 1)). (**C**) Densitometric analysis of zymograms from multiple separate experiments (*n*=4) showing MMP-9 expression by VB6 cells on BSA, LAP following incubation with an irrelevant control antibody (Ctl; W632 (anti-MHC class 1)) and LAP following blockade with anti-αvβ6 antibody (10D5). Results are expressed relative to MMP-9 expression by VB6 cells on BSA-coated plastic (=100). Error bars represent standard deviation. These data suggest that αvβ6-LAP interaction modulates MMP-9 expression in VB6 cells.

Expression of MMP-9 on BSA-, fibronectin-, and LAP-coated tissue culture plastic was examined by zymography. Zymography on supernatant samples from cells grown in serum-free medium showed that the cells produced predominantly MMP-9 and that this enzyme was mainly present in proenzyme form. Similar to the effect observed on fibronectin (Thomas *et al*, 2001c), VB6 cells showed significant upregulation of MMP-9 when plated on LAP (*P*=<0.001; Figure 4A,C

). This upregulation could be inhibited by prior incubation of VB6 cells with the specific anti-αvβ6 blocking antibody, 10D5 (*P*=<0.001; Figure 4B,C). No upregulation of MMP-9 on LAP was observed in the C1 control cells (*P*=0.134; data not shown). These data confirm that, similar to fibronectin, LAP interaction with the αvβ6 integrin produces an upregulation of MMP-9.

### Soluble LAP inhibits adhesion and migration of VB6 cells to fibronectin

**Figure 5:**
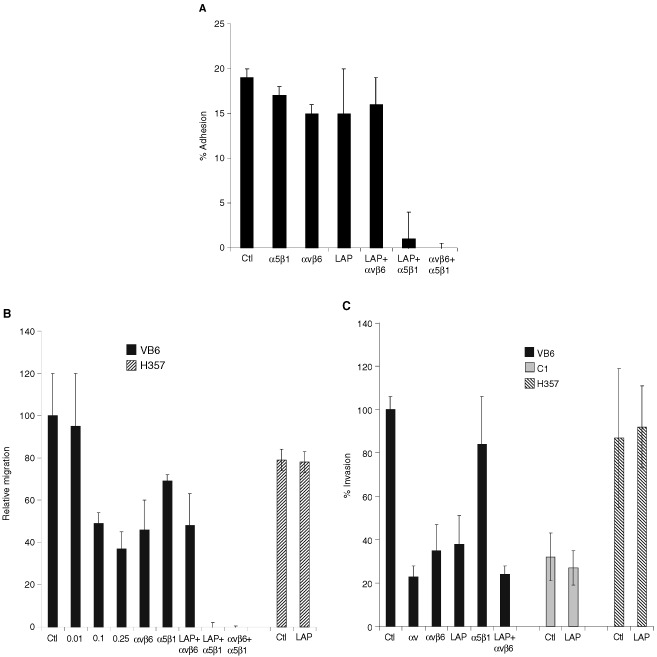
Soluble LAP inhibits αvβ6 interaction with fibronectin. (**A**) LAP inhibits VB6 adhesion to fibronectin. Chromium [^51^Cr]-labelled VB6 cells were added to fibronectin-coated 96-well plates containing an irrelevant control antibody (W632) or test antibodies against αvβ6 (10D5), α5β1 (P1D6) and LAP (0.25 μg ml^−1^) or combinations thereof. Figure shows a representative experiment performed in quadruplicate. Results are expressed relative to binding in the presence of a control antibody (=100). Background binding to BSA has been subtracted from the results. Error bars represent standard deviation. VB6 cells adhere to fibronectin through αvβ6 and α5β1 and a combination of antibodies against both integrins is required to block adhesion completely (Thomas *et al*, 2001b). Treating the cells solely with LAP, or antibodies against αvβ6 or α5β1 did not inhibit adhesion significantly (neither did a combination of LAP and anti-αvβ6). However, combinations of anti-α5β1 and LAP or anti-αvβ6 and anti-α5β1 inhibited adhesion completely. These data confirm that soluble LAP produces a similar effect to anti-αvβ6 antibody and suggests that LAP may alter keratinocyte binding to fibronectin through its interaction with the αvβ6 integrin. (**B**) LAP inhibits VB6 migration to fibronectin. To determine the effect of LAP on cell migration, the cells were treated with varying concentrations of LAP peptide prior to plating and then allowed to migrate for 8 h before counting. A representative experiment performed in quadruplicate is shown. Results are expressed relative to migration in the presence of a control antibody (=100). Error bars represent standard deviation. Haptotactic migration of VB6 cells towards fibronectin is modulated through αvβ6 and α5β1 integrins and maximal inhibition of migration is produced by blocking both integrins (Thomas *et al*, 2001b). Migration of VB6 cells towards fibronectin was inhibited by a concentration of LAP as low as 0.1 μg ml^−1^ (51% inhibition). At higher concentrations a further degree of inhibition was seen (0.25 μg ml^−1^=63% inhibition). This level of inhibition was similar to that produced by anti-αvβ6 antibody (54% inhibition) and did not differ significantly from a combination of LAP and anti-αvβ6 antibody (52% inhibition). Complete inhibition of migration was produced using a combination of soluble LAP with anti-α5β1 antibody (or a combination of anti-αvβ6 with anti-α5β1 antibody). Soluble LAP had no effect of the migration of H357 αv-negative cells. These data confirm that soluble LAP produces a similar effect to anti-αvβ6 antibody and suggest that LAP may inhibit αvβ6-dependent keratinocyte migration towards fibronectin. (**C**) LAP inhibits αvβ6-dependent invasion through Matrigel. Cell invasion assays were performed over 72 h using Matrigel coated polycarbonate filters. To assess the effect of soluble LAP invasion, cells were treated with LAP (0.5 μg ml^−1^) for 30 min at 4°C prior to plating. Following incubation the cells in the lower chamber (including those attached to the undersurface of the membrane) were trypsinised and counted on a Casy 1 counter (Sharfe System GmbH, Germany). Figure shows a representative experiment performed in quadruplicate. Error bars represent standard deviation. Pre-incubation of VB6 cells with soluble LAP inhibited invasion (62% inhibition), producing a similar level of inhibition to anti-αvβ6 antibody (58% inhibition). Soluble LAP had no effect on invasion of H357 αv-negative cells or C1 control cells which express low levels of endogenous αvβ6. These data suggest that soluble LAP may inhibit αvβ6-dependent cell invasion.

VB6 cells bind to fibronectin through both αvβ6 and α5β1 integrins such that to completely inhibit adhesion to this substrate, a combination of blocking antibodies against both integrins is required (Thomas *et al*, 2001b). To determine whether LAP competitively inhibits αvβ6-dependent binding to fibronectin, VB6 cells were incubated with LAP (0.25 μg ml^−1^) or antibodies against αvβ6 (10D5) or α5β1 (P1D6) prior to plating. Treating the cells solely with LAP, or antibodies against αvβ6 or α5β1 did not inhibit adhesion to fibronectin significantly (Figure 5A

). Similarly, a combination of LAP and anti-αvβ6 produced no significant effect. However, a combination of anti-α5β1 and LAP (or a combination of anti-α5β1 and anti-αvβ6) completely inhibited adhesion to fibronectin (Figure 5A). These data are consistent with the possibility that soluble LAP may alter keratinocyte binding to fibronectin through its interaction with the αvβ6 integrin.

Haptotactic migration of VB6 cells towards fibronectin is also modulated through αvβ6 and α5β1 integrins (Thomas *et al*, 2001b). To determine whether LAP inhibits αvβ6-dependent migration towards fibronectin, VB6 cells were incubated with varying concentrations of LAP prior to plating. A titration of LAP concentrations showed that migration towards fibronectin could be inhibited by a concentration of LAP as low as 0.1 μg ml^−1^ (51% inhibition; *P*=0.001; Figure 5B). At higher concentrations a further degree of inhibition was seen (0.25 μg ml^−1^=63% inhibition; *P*=0.001; Figure 5B). This inhibition was similar to that produced by anti-αvβ6 antibody alone or a combination of LAP and anti-αvβ6 antibody (46 and 48% inhibition respectively; Figure 5B). Complete inhibition of migration was produced using a combination of LAP with anti-α5β1 antibody or (as demonstrated previously), a combination of anti-α5β1 and anti-αvβ6 antibodies (Thomas *et al*, 2001b). Migration of αv-negative H357 cells towards fibronectin was not affected by the presence of LAP in the assay. These data suggest that LAP may inhibit αvβ6-dependent keratinocyte migration towards fibronectin.

### Soluble LAP inhibits invasion of VB6 cells through Matrigel

VB6 cells show increased αvβ6-dependent invasion through Matrigel relative to C1 control cells (Thomas *et al*, 2001b,c). To determine whether LAP inhibits αvβ6-dependent invasion, VB6 cells were incubated with LAP (0.5 μg ml^−1^) or antibodies against the αv subunit, αvβ6 or α5β1. Figure 5C confirms that prior incubation of VB6 cells with LAP inhibits invasion by VB6 cells (62% inhibition; *P*=<0.001), producing a similar level of inhibition to that obtained with anti-αvβ6 antibody (58% inhibition). Invasion of H357 cells (which invade at a similar level to VB6 cells through non-αvβ6 mechanism) or C1 cells was not affected by LAP. In addition, to confirm that cell invasion was not affected by endogenously secreted TGF-β1 or by TGF-β1 present within serum, we repeated the assays incorporating a TGF-β1 blocking antibody or TGF-β1 soluble receptor. Neither of these TGF-β inhibitors produced an effect on invasion by VB6 cells (data not shown). These data confirm that soluble LAP may specifically inhibit αvβ6-dependent cell invasion.

## DISCUSSION

*De novo* expression of αvβ6 is seen in a number of epithelial malignancies, particularly oral squamous cell carcinoma (SCC) (Breuss *et al*, 1995; Jones *et al*, 1997; Hamidi *et al*, 2000; Ahmed *et al*, 2002). Expression of this integrin is often higher at the invasive front, supporting the contention that αvβ6 is capable of playing an active role in tumour progression. We have shown previously that over-expression of αvβ6 in oral SCC cells promotes cell migration, invasion and MMP-9 expression (Thomas *et al*, 2001b, c). Moreover, recent data from Xue *et al* (2001) demonstrated that inhibition of αvβ6 could significantly retard the growth of human SCC cells implanted orthotopically into athymic mice. These studies provide strong evidence that the activity of αvβ6 promotes development of SCC and that this integrin may represent a possible therapeutic target in the treatment of SCC. It is therefore essential that we gain a complete understanding of how interaction of αvβ6 with its immediate environment can result in changes in cell behaviour.

The principal extracellular matrix ligands for αvβ6 are fibronectin and tenascin, although αvβ6 also modulates migration on vitronectin (Busk *et al*, 1992; Prieto *et al*, 1993; Huang *et al*, 1998). All three ligands are found in relative abundance at sites of tumour growth (Mighell *et al*, 1997; Johnsen *et al*, 1998; Ramos *et al*, 1998; Kosmehl *et al*, 1999). Recently it has been shown that αvβ6 binds to and activates latent TGF-β1 and TGF-β3 via an interaction with its pro-peptide, the latency-associated peptide (LAP) (Munger *et al*, 1999; Annes *et al*, 2002). Since changes in the local concentration of TGF-β1/LAP occur naturally during SCC, we investigated whether LAP could modulate αvβ6-dependent activities and thus the biological behaviour of transformed keratinocytes.

The role of TGF-β1 in SCC is complex. Studies suggest that TGF-β1 has biphasic actions on tumour cells, having an important negative growth effect in the early stages of carcinogenesis, but at later stages enhancing invasion and metastasis through epigenetic mechanisms (Akhurst and Balmain, 1999; Akhurst and Derynck, 2001). Recent data suggests that aberrant activation of MAPK pathways may play an important role in diverting the TGF-β response towards a pro-oncogenic outcome, and that TGF-β and activated Ras may cooperate to promote invasive, metastatic disease (Park *et al*, 2000). However, Ras mutations are uncommon in SCC in the western world (Rumsby *et al*, 1990; Yeudall *et al*, 1993). Moreover, although the parental cell line H357, used in this study, harbours two point mutations in the c-Ha-ras gene (Yeudall *et al*, 1993) it retains its sensitivity to the growth inhibitory effects of TGF-β1 (Paterson *et al*, 1995). In fact, the majority of studies have found that oral SCC cell lines are growth inhibited by TGF-β1 (Paterson *et al*, 1995; Malliri *et al*, 1996). A recent study has shown that TGF-β-dependent growth inhibition of oral SCC cells may occur in the absence of Smad-4, a key molecule in TGF-β signalling (Akhurst and Derynck, 2001; Derynck *et al*, 2001), suggesting that other signalling pathways may also mediate growth inhibitory responses (Paterson *et al*, 2002). Several mechanisms by which oral SCC may escape the negative growth regulation of TGF-β include producing less autocrine TGF-β1, expressing low levels of the receptor TβR-II or losing expression of Smad-2 (Fahey *et al*, 1996; Muro-Cacho *et al*, 2001; Paterson *et al*, 2001). Although structural defects in the TβR-I and TβR-II receptors have been reported in oral SCC, these are not common. These data suggest that alterations of gene expression are likely to be more important than mutations in the pathogenesis of oral SCC (Garrigue-Antar *et al*, 1995; Wang *et al*, 1997; Paterson *et al*, 2001). TGF-β is frequently detectable in SCC, particularly in the more advanced stages of tumour progression, and relatively high concentrations of TGF-β are usually found in tumour stroma (reviewed by Pasche, 2001). Thus there is a significant likelihood that TGF-β could affect SCC tumour cell behaviour via integrin-dependent interaction with its pro-peptide, LAP, independent of classical TGF-β signalling.

We first determined how keratinocyte-derived cells adhered to LAP. It has been reported that in addition to αvβ6, αvβ5 and αvβ1 can also serve as receptors for LAP (Munger *et al*, 1998). We report here that although the oral SCC cells used in this study express two of these integrins (αvβ6 and αvβ5), these keratinocyte-derived cells bind and migrate towards LAP solely via αvβ6. These findings were confirmed using primary keratinocytes (which express αvβ6 and αvβ5 *in vitro*), which also bind to and migrate toward LAP solely through αvβ6 (data not shown). The oral SCC line, VB6, exhibited haptotactic migration toward LAP, although at significantly lower levels than VB6 migration towards fibronectin. Interestingly, αvβ6-dependent cell invasion of VB6 cells was observed when serum was replaced with LAP (or fibronectin) in the lower chamber of the invasion assay. It would seem that these matrix proteins act as chemoattractants for αvβ6-dependent invasion. LAP also induced an αvβ6-dependent upregulation of pro-MMP-9 expression, as was previously shown on fibronectin (Thomas *et al*, 2001a,c). It appears therefore that LAP modulates similar αvβ6-dependent functions as fibronectin.

The interaction between αvβ6 and TGF-β in SCC appears complex. It is possible that αvβ6 may activate TGF-β1 and produce a growth inhibitory effect, whereas, depending on whether LAP is immobilised in the ECM or present as a soluble ligand, it is also possible that LAP could modulate both cell movement and protease production. The net effect on tumour behaviour may depend on the stage of tumour development. Growth inhibition may be dominant in the earlier stages of carcinogenesis with TGF-β1 acting as a tumour suppressor. However, in the later stages of tumour development, as cells become refractory to growth inhibition, then the role of LAP in promoting αvβ6-dependent cell movement and MMP-9 expression may assume greater significance, and this may be one of the ways in which TGF-β promotes tumour development.

In summary, previously we reported that it was likely that αvβ6 promoted oral SCC invasion by increasing migration, pro-MMP9 production and invasion (Thomas *et al*, 2001b,c). In this study we now show that these αvβ6-dependent processes of oral SCC cells are likely to be modulated *in vivo* by ligation to a high-affinity, αvβ6-specific ligand for oral SCC cells, the TGFβ-associated pro-peptide, LAP.
